# Slow repair of lipid peroxidation-induced DNA damage at p53 mutation hotspots in human cells caused by low turnover of a DNA glycosylase

**DOI:** 10.1093/nar/gku520

**Published:** 2014-07-31

**Authors:** Jordan Woodrick, Suhani Gupta, Pooja Khatkar, Sanchita Sarangi, Ganga Narasimhan, Akriti Trehan, Sanjay Adhikari, Rabindra Roy

**Affiliations:** Department of Oncology, Lombardi Comprehensive Cancer Center, Georgetown University Medical School, Washington, DC 20057, USA

## Abstract

Repair of oxidative stress- and inflammation-induced DNA lesions by the base excision repair (BER) pathway prevents mutation, a form of genomic instability which is often observed in cancer as ‘mutation hotspots’. This suggests that some sequences have inherent mutability, possibly due to sequence-related differences in repair. This study has explored intrinsic mutability as a consequence of sequence-specific repair of lipid peroxidation-induced DNA adduct, 1, *N*^6^-ethenoadenine (εA). For the first time, we observed significant delay in repair of ϵA at mutation hotspots in the tumor suppressor gene p53 compared to non-hotspots in live human hepatocytes and endothelial cells using an in-cell real time PCR-based method. In-cell and *in vitro* mechanism studies revealed that this delay in repair was due to inefficient turnover of *N*-methylpurine-DNA glycosylase (MPG), which initiates BER of εA. We determined that the product dissociation rate of MPG at the hotspot codons was ≈5–12-fold lower than the non-hotspots, suggesting a previously unknown mechanism for slower repair at mutation hotspots and implicating sequence-related variability of DNA repair efficiency to be responsible for mutation hotspot signatures.

## INTRODUCTION

Genomic instability is a major ‘enabling’ hallmark of cancer cells. Via genetic and epigenetic changes, cells acquire functional traits which promote survival, unregulated proliferation and migration. Multiple DNA repair pathways are a major barrier to genomic instability, thus DNA repair enzymes function as tumor suppressor genes (1). Usually these repair enzymes ensure that damaged DNA is not erroneously copied by the cellular replication machinery, preventing mutations in critical genes that could lead to genomic instability. Interestingly, mutations observed in human cancers are often at ‘mutation hotspots’ which suggests that either the resulting phenotypic change from those particular codons is more advantageous than at other positions or that some DNA sequences have inherent mutability. The present study was undertaken to elucidate sequence context effects on DNA repair enzymes, specifically the intrinsic mutability of specific hotspot codons in the tumor suppressor gene p53.

DNA repair pathways have a particularly important role in carcinogenesis associated with chronic inflammatory premalignant conditions, such as hepatitis, pancreatitis and colitis. These conditions are often associated with accumulation of oxidative DNA damage due to excess free radicals, including reactive oxygen species and DNA-reactive aldehydes from lipid peroxidation (LPO) (2). LPO products can react with all four bases to form exocyclic adducts, one of which is an adenine adduct, 1, *N*^6^-ethenoadenine (εA) (3). Ethenoadenine is specifically recognized and excised by *N*-methylpurine-DNA glycosylase (MPG) to initiate the base excision repair (BER) pathway (4,5). The presence and accumulation of εA have been demonstrated in a variety of chronic inflammatory conditions that carry increased risk of cancer, including hemochromatosis and Wilson's disease, chronic pancreatitis, Crohn's disease, ulcerative colitis and familial adenomatous polyposis (6). Additionally, high levels of εA were observed in esophageal cells in patients with upper aerodigestive tract cancers associated with long-term alcohol abuse and smoking (7). Ethenoadenine repair has also been shown to be abrogated in inflammation-mediated lung adenocarcinoma (8). Additionally, some environmental carcinogens, such as vinyl chloride (VC), lead to εA accumulation in several organs (9). In both bacterial and mammalian experimental systems, unrepaired εA has been shown to cause all types of base substitutions (10,11). Importantly, VC-induced liver malignancies, as well as other aforementioned cancers associated with accumulation of εA during a premalignant inflammatory state, harbor adenine mutations at hotspot codons, including codons 249, 179 and 255 in the p53 gene (12–16).

Sequence context-specific repair by MPG and other BER proteins has been examined previously in other studies. Repair by 8-oxoguanine DNA glycosylase (OGG1), a DNA glycosylase that excises an oxidative guanine adduct, 8-oxo-dG, was shown to be affected by flanking modified bases and mismatches but not neighboring unmodified bases (17). On the other hand, the presence of an A:T base pair 5′ to 8-oxo-dG reduced the excision activity of *Escherichia coli* formamidopurine DNA glycosylase (MutM), which is the bacterial enzyme that excises 8-oxo-dG, by 3-fold (18). Apurinic/apyrimidinic endonuclease-1 (APE1) also showed some sequence context-dependent differences in incision activity but without any pattern to distinguish ‘efficient’ versus ‘inefficient’ sequences (19). MPG exhibited sequence context-dependent efficiency of hypoxanthine (Hx) removal and was shown to excise εA more efficiently when the damaged base was flanked by GC-rich sequences compared to AT-rich sequences (20–22). Additionally, removal of εA by mouse Mpg was shown to be somewhat affected by neighboring A:T base pair stacking (23). One previous study examined strand-specific repair of alkylation adducts in a biologically relevant manner, focusing on strand-bias (transcribed versus non-transcribed) of MPG-induced repair of adducts within a gene of interest (24).

The aforementioned studies documented a role for the observed differences in BER enzyme activity at different DNA sequences in mutagenesis, but most of the sequences utilized in the activity experiments were not biologically relevant, and no in-cell or *in vivo* studies were conducted to confirm the *in vitro* results. Additionally, it might be suggested that mutations arise in some DNA sequences more often than others because of a difference in adduct accumulation at different sites in the genome. However, an adduct accumulation experiment with chloroacetaldehyde (CAA), a metabolite of VC, showed εA adduct formation at hotspot codons 249 and 255 as well as some non-hotspot codons such as 247 at an even higher level (25). Then the mechanism behind mutations at hotspot codons 249 and 255 may not depend simply on adduct formation on those sites alone. The persistence of DNA adduct rather than the rate of adduct formation would lead to mutations as a result of erroneous DNA replication. It is plausible that a low repair rate of εA at certain sequences may contribute to higher accumulation of εA and subsequent mutations at specific codons. However, the repair rate of εA has not been directly compared at hotspot codons 179, 249 and 255 versus non-hotspot codons in p53. Thus, the objective of this study is to examine and compare the repair kinetics of εA at mutation hotspots and non-hotspot codons of the p53 gene in-cell and *in vitro* using the mutation hotspot patterns observed in cancers associated with accumulation of εA during a premalignant inflammatory state as a model. Our study focuses on the sites of interest (mutation and non-mutation sites) in a codon-specific manner, hypothesizing that repair of εA at frequently mutated codons in p53 is repaired less efficiently than εA at non-mutation sites in p53. Codons 246 and 247 were chosen as representative non-hotspot codons since they harbored adenine mutations in <2% of tumors with the highest rate of adenine mutations in the IARC TP53 database, including liver, esophageal and colorectal cancers, among others (16) (Supplementary Figure S1A and B). Our approach included in-cell repair analysis by a modified real time PCR-based assay in human hepatic and endothelial cells followed by a comprehensive mechanism analysis *in vitro.*

## MATERIALS AND METHODS

### M13mp18-p53 phagemid construction and single-stranded DNA (ssDNA) isolation

The p53 cDNA sequence was cloned from pcDNA4/TO-p53 (a gift from Dr Maria Laura Avantaggiati, Georgetown University) using primers containing the HindIII and XbaI restriction sites on the 5′ and 3′ ends, respectively. The p53 cDNA was cloned into M13mp18 phagemid vector at the HindIII and XbaI sites so that the M13mp18-p53 phagemid (+) strand contained p53 in the (−) orientation. The phage suspension was prepared following a standard method (26). Single-stranded M13mp18-p53 DNA was isolated from the phage suspension using the Qiaprep Spin M13 kit (Qiagen, Gaithersburg, MD, USA).

### εA-M13mp18-p53 and APsite-M13mp18-p53 *in vitro* construct preparation

The εA-M13mp18-p53 *in vitro* construct preparation included three main steps: phoshorylation of the εA-containing primer oligonucleotide, annealing of the oligonucleotide to the ssDNA, and the primer extension reaction. Each of these steps is described in detail in the Supporting Information.

### Cell culture

HepG2 cells and immortalized mouse embryonic fibroblasts (MEFs) were cultured in DMEM (Mediatech, Manassas, VA, USA) with 10% fetal bovine serum (FBS) and 1% penicillin–streptomycin (Mediatech, Manassas, VA, USA). *Mpg*^+/+^ and *Mpg*^−/−^ MEFs were a gift from Dr Leona Samson, Massachusetts Institute of Technology (27). HUVECs were cultured in Ham's F-12K (Kaighn's) Medium (Life Technologies, Grand Island, NY, USA) supplemented with 10% FBS, 1% penicillin–streptomycin, 0.1 mg/ml heparin (Sigma, St Louis, MO, USA) and 0.03 mg/ml endothelial cell growth supplement (ECGS) (Sigma, St Louis, MO, USA).

### In-cell repair of εA and AP-sites in human cells

**Figure 1.**
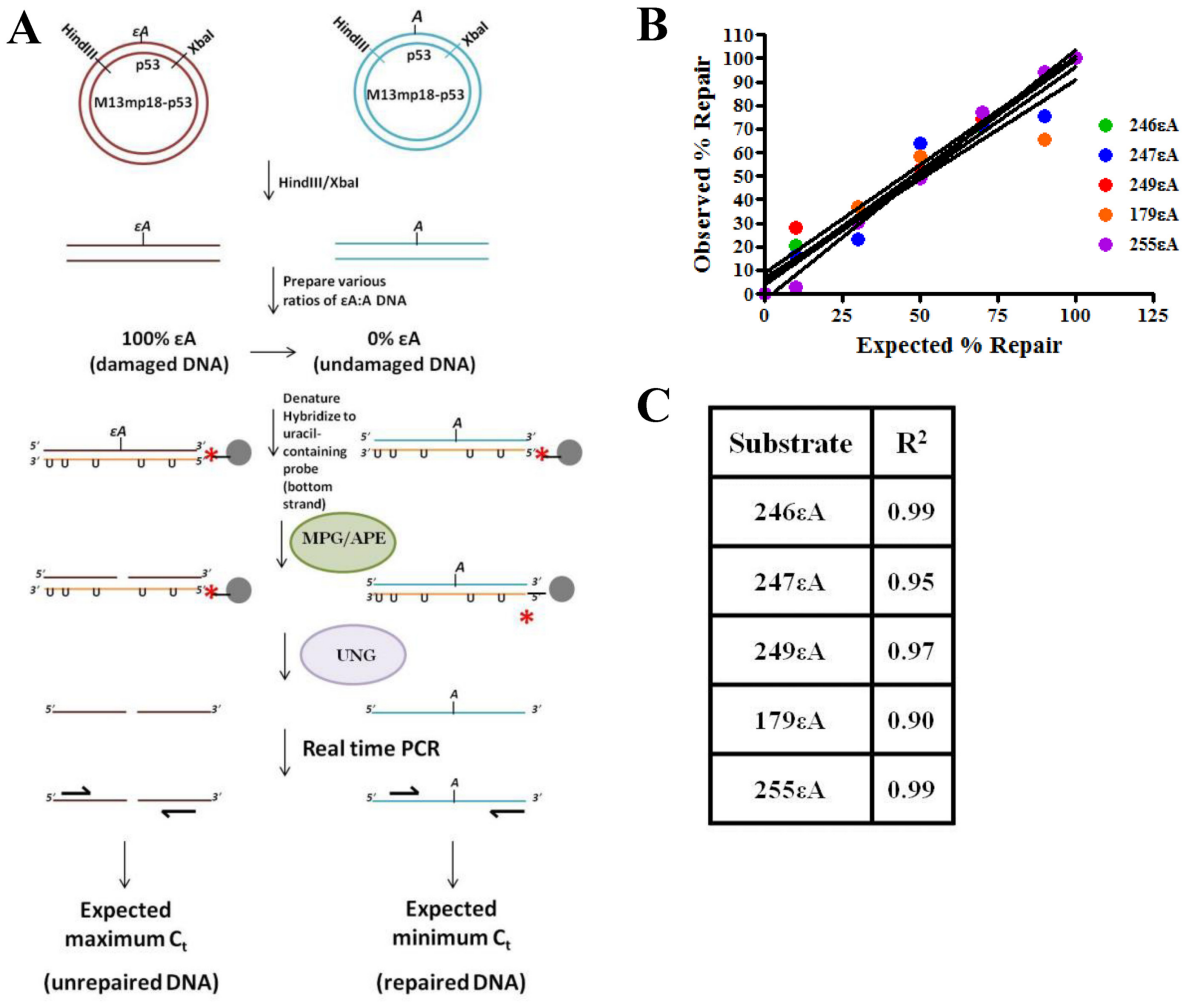
Experimental design and method validation by mixing experiment *in vitro*. (**A**) Schematic representation of repair assay. (**B**) 246εA, 247εA, 249εA, 179εA and 255εA mixing experiment demonstrating the linearity of the assay. (**C**) Table reports the *R*^2^ values of the trendline for each mixing experiment graphed in (B).

**Figure 2.**
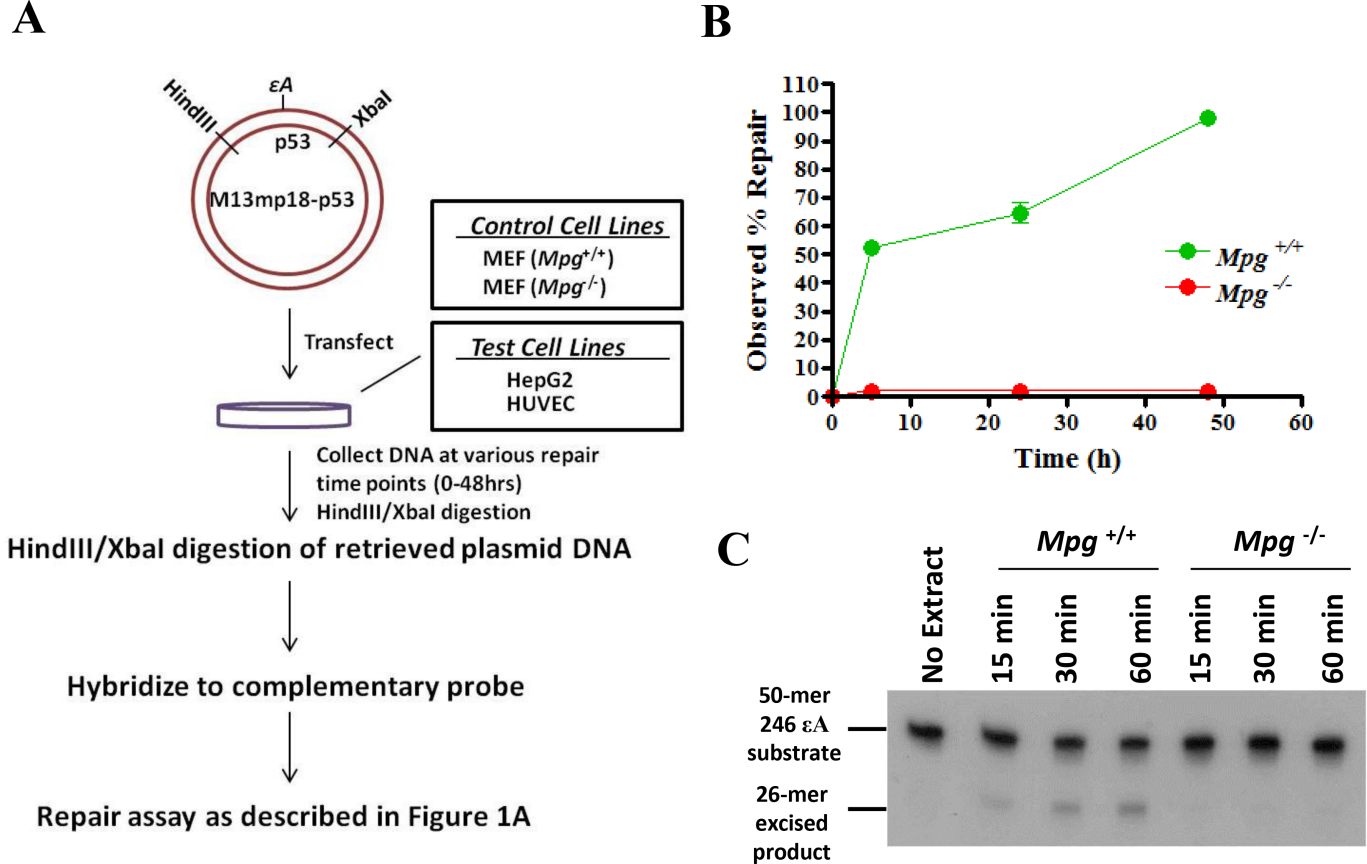
Experimental design and method validation in cells. (**A**) Schematic representation of in-cell repair assay. (**B**) Repair of 246εA in wild-type (*Mpg*^+/+^) and *Mpg*^−/−^ MEFs as measured by real time PCR. (**C**) MPG excision activity on 246εA oligonucleotide substrate in *Mpg*^+/+^ and *Mpg*^−/−^ MEF nuclear extract. Reactions were incubated at 37°C for 15, 30 and 60 min before termination at 65°C.

In order to detect and quantify repair of εA or AP-sites in human cells, plasmids were first transfected into human cells and retrieved at various time points post-transfection. After harvesting the episomal DNA from the cells, the DNA was digested with HindIII and XbaI restriction enzymes to isolate the p53 insert, then hybridized to a complementary uracil-containing full length p53 probe. Samples were subsequently digested with MPG and/or APE1 to convert εA- or AP-site-containing plasmid, respectively, to nicked DNA. Finally, the samples were treated with uracil DNA glycosylase to digest the complementary probe strand, then repair was determined by real time PCR using primers flanking the damage site. Each of these steps is described in detail in the Supporting Information and illustrated in Figures 1A and 2A.

### MPG-mediated excision activity assay

Purified MPG (4–8 nM) was incubated with 7 nM sequence-specific εA-containing ^32^P-labeled oligonucleotide substrates (see Supplementary Table S1 for oligonucleotide substrate sequences) for 10 min at 37°C in an assay buffer containing 25 mM HEPES, pH 7.9, 150 mM NaCl, 100 μg/ml bovine serum albumin (BSA), 0.5 mM dithiothreitol (DTT) and 10% glycerol in a total volume of 20 μl. The MPG reactions were subsequently stopped at 65°C for 10 min then cooled to room temperature for 15 min. AP-sites were cleaved with reaction mixture of 15 nM APE1 and 5 mM MgCl_2_ at 37°C for 15 min. Reactions were diluted 1:1 with a loading buffer containing 1× gel loading dye (New England Biolabs, Ipswich, MA, USA), 85% formamide, and 0.3N NaOH. Samples were subsequently heated at 95°C for 3 min followed by cooling on ice for 3 min. Samples were resolved by electrophoresis at 60°C using Criterion gel cassettes (BioRad, Hercules, CA, USA) containing 20% polyacrylamide (BioRad, Hercules, CA, USA) and 7M urea (BioRad, Hercules, CA, USA). Radioactivity was quantified by exposing the gel to X-ray films and quantifying the band intensities using an imager (Chemigenius Bioimaging System, Frederick, MD, USA) and software (GeneTool, Syngene Inc., San Diego, CA, USA). Reactions using nuclear extracts were performed similarly, using 2 μg of HepG2 nuclear extract and 1 nM sequence-specific εA-containing ^32^P-labeled oligonucleotide substrates for 10 and 20 min at 37°C. Reactions with 5 μg MEF nuclear extract were performed similarly at 37°C for 15, 30 and 60 min.

### Binding studies using surface plasmon resonance

Binding studies were performed in a Biacore T100 system (Biacore, Uppsala, Sweden) as described previously with some modifications (28). 50-mer oligonucleotides containing εA in the sequence context of codons 246, 247, 249, 179 and 255 (see Supplementary Table S1 for sequences of oligonucleotides) were biotinylated and immobilized on streptavidin-coated C1 Biacore chips. Then binding parameters of MPG were determined using 0–40 nM protein in a binding buffer containing 10 mM HEPES–KOH, pH 7.6, 90 mM KCl and 0.05% surfactant P20 (Biacore, Uppsala, Sweden) at 7°C. Injections, regeneration and kinetics analysis were performed as described previously (28). Kinetic parameters for binding were calculated using a 1:1 binding model and Langmuir isotherm based on the on/off rates and protein concentrations for each oligonucleotide substrate using BiaEvaluation software (Biacore, Uppsala, Sweden).

### Single turnover (STO) kinetic study

The kinetic studies were carried out and analyzed as described previously (28). Typically, 500 nM MPG was incubated with 1 nM of 5′-^32^P-labeled codon-specific εA-containing oligonucleotide substrate (see Supplementary Table S1 for oligonucleotide sequences) at 37°C in MPG excision activity assay buffer in a total volume of 100 μl. Aliquots of 5 μl were heat-inactivated at 80°C in pre-heated microcentrifuge tubes at various time points from 0 to 10 min. AP-site cleavage and sample denaturation and resolution by gel electrophoresis were performed as described in the excision activity assay. Data were analyzed using the equation:

\begin{equation*} [P] = A_0 \{ 1 - \exp ( - k_{{\rm obs}} t)\} \end{equation*}

where *A*_0_ represents the amplitude of the exponential phase and *k*_obs_ is the observed rate constant of the reaction, which is approximately equivalent to *k*_chem_, the rate of glycosidic bond cleavage.

### Burst analysis

Reactions were carried out as described previously (28). Briefly, 7 nM MPG was incubated with 75 nM of 5′-^32^P-labeled codon-specific εA- or Hx-containing oligonucleotide substrate for different lengths of time (0–10 min) at 37°C under conditions similar to those described in the single turnover (STO) kinetic study. The titration experiments were carried out in a similar manner with 3.5, 7, 10 and 20 nM MPG. The data were analyzed according to the equation:

\begin{equation*} [P] = A_0 \{ 1 - \exp ( - k_{{\rm obs}} t)\} + k_{{\rm ss}} t \end{equation*}

where *k*_ss_ is the slope of the linear phase, which is the turnover rate, and *A*_0_ represents the amplitude of the burst. The rate of product dissociation (*k*_pd_) is estimated by the ratio of *A*_0_ over *k*_ss_.

### Active site titration

Reactions were carried out as described for the burst analysis, using 75 nM of 5′-^32^P-labeled codon-specific εA- or Hx-containing oligonucleotide substrate and 3.5, 7, 10 and 20 nM MPG. Fitting the data to the equation above (for burst analysis) provides an accurate value for the amplitude of the burst and the active enzyme concentration by extrapolation of the linear phase of the reaction to determine the *y* intercept.

## RESULTS

### Real time PCR-based repair assay development and validation *in vitro* and in-cell

In order to monitor p53 codon-specific repair of εA in live cells, the p53 cDNA sequence was cloned into the M13mp18 phagemid (see ‘Materials and Methods’ section). Since the codons of interest were not in close proximity to exon–intron junctions, the cDNA sequence was used for practical purposes and deemed representative of the local sequence architecture for each codon in the context of chromatin. The single stranded DNA from this M13mp18-p53 construct was used to generate plasmids with a single εA placed at codons of interest. In order to validate the real time PCR-based method of monitoring in-cell repair of εA at mutation hotspot and non-hotspot codons in the p53 gene, a linear mixing experiment was performed to mimic a range of repair from 0 to 100%. M13mp18-p53 phagemids containing a single εA at codon 246, 247, 249, 179 or 255 were mixed with different amounts of undamaged M13mp18-p53, which represented the repaired DNA (Figure 1A). These seven standards were then assayed for repair, and the results were plotted against the expected repair values, such as 0, 10, 30, 50, 70, 90 and 100% based on the ratio of εA-DNA to undamaged DNA (Figure 1B). At each codon, the observed repair increased linearly with increasing amounts of undamaged DNA (*R*^2^ = 0.90–0.99) (Figure 1C). To determine the validity of the assay in cells, M13mp18-p53 phagemid containing εA at codon 246 (246εA) and undamaged plasmid were transfected into *Mpg*^+/+^ and *Mpg*^−/−^ MEFs (Figure 2A). Beginning 5 h after transfection (0 h), the plasmid DNA was harvested at various time points (5, 16, 24 and 48 h) and assayed for repair of εA at codon 246. As expected, no repair of εA was detected in the *Mpg*^−/−^ cells up to 48 h post-transfection, while 50% of εA was repaired in <5 h (*t*_1/2_ < 5 h) in the *Mpg*^+/+^ cells (Figure 2B). The *Mpg* status of the MEFs was verified by *Mpg* excision activity assay using cell-free extracts, where excision of the 246εA oligonucleotide substrate was observed in *Mpg^+^*^/+^ cell extract but not *Mpg*^−/−^ cell extract (Figure 2C). It should be noted that another non-BER mechanism of εA repair exists in human cells, which has been shown to be initiated by both human AlkB homologues 2 and 3 (ABH2 and ABH3) (29–31). This mode of εA repair proceeds via the direct reversal pathway, but previous studies have demonstrated that MPG is the major repair protein for the removal of εA (27,32). Therefore, we did not consider the contribution of ABH2- and ABH3-mediated repair in the interpretation of the results of this study.

### Repair of εA at mutation hotspots is significantly delayed in both hepatocytes and endothelial cells

**Figure 3.**
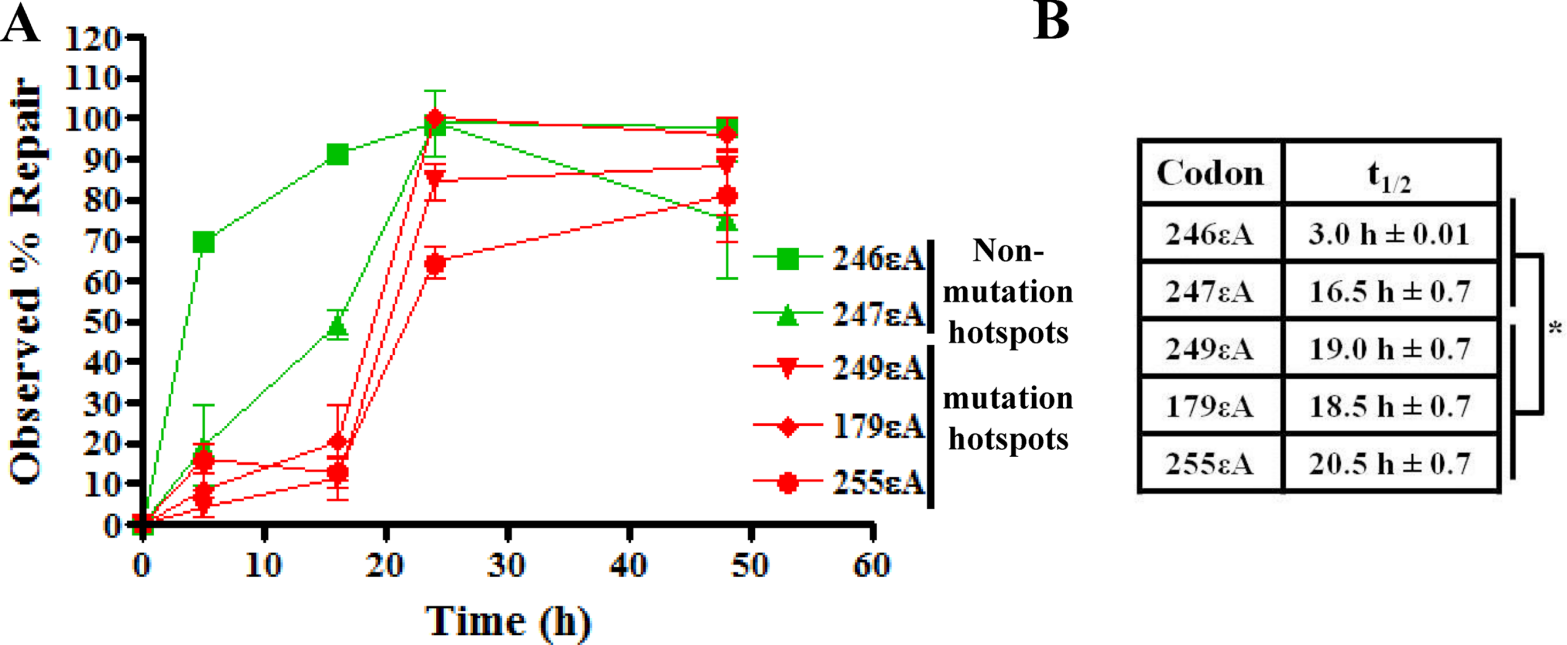
Slow repair of εA at mutation hotspot codons in p53 in HepG2 cells. The real time PCR-based method for quantifying repair of εA in cells was utilized to quantify repair of εA at mutation hotspots and non-mutation hotspots in the p53 gene in hepatocytes. (**A**) Repair of εA at different codons in p53 in HepG2 cells as detected by real time PCR. Green denotes non-mutation hotspot codons and red denotes mutation hotspots. For each graphed point, *N* = 3, and error bars denote SEM. (**B**) Table reports *t*_1/2_, indicating the length of time required for 50% repair of εA. *P-value < 0.05, unpaired two-tailed Student's *t*-test comparing all non-hotspot *t*_1/2_ values with all hotspot *t*_1/2_ values.

**Figure 4.**
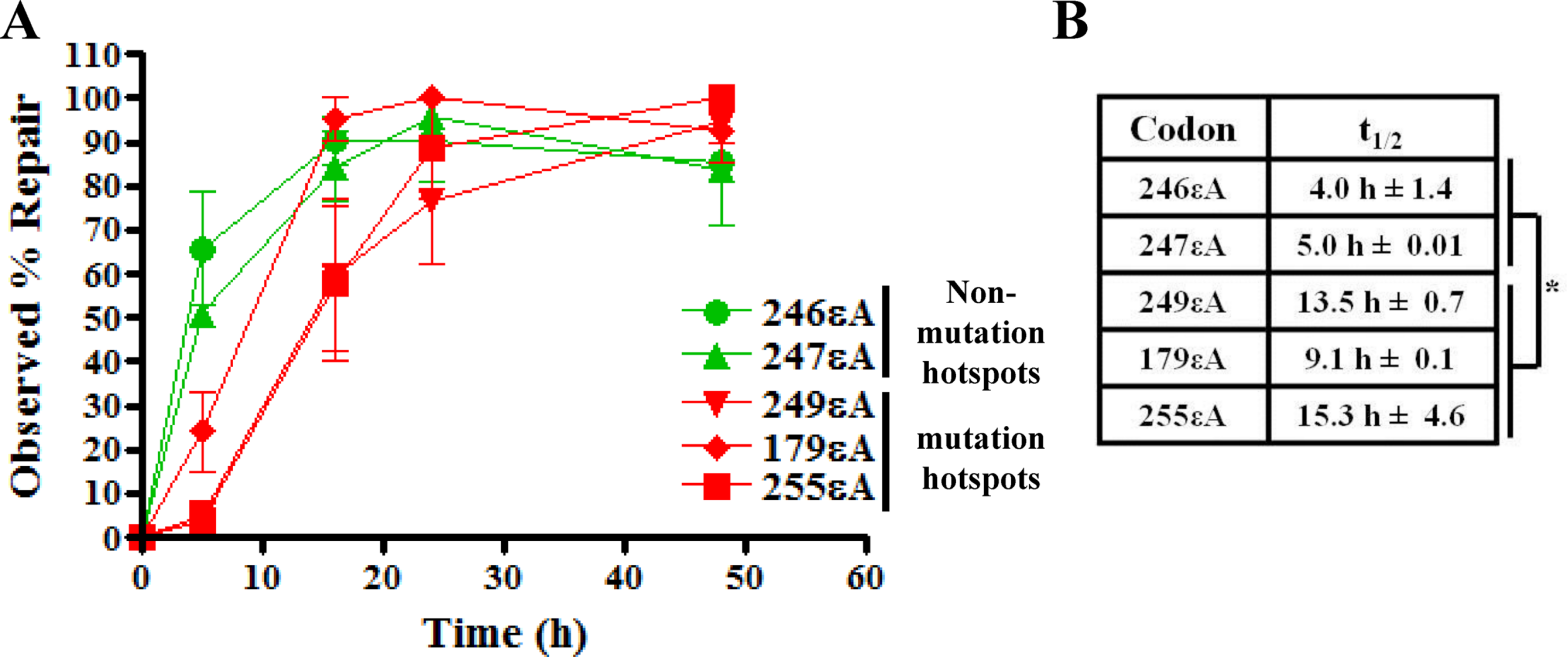
Slow repair of εA at mutation hotspot codons in p53 in HUVECs. (**A**) The real time PCR-based method for quantifying repair of εA in cells was utilized to quantify repair of εA at mutation hotspots and non-mutation hotspots in the p53 gene in endothelial cells. For each graphed point, *N* = 3, and error bars denote SEM. (**B**) Table reports *t*_1/2_, indicating the length of time required for 50% repair of εA. *P-value < 0.05, unpaired two-tailed Student's *t*-test comparing all non-hotspot *t*_1/2_ values with all hotspot *t*_1/2_ values.

In HepG2 cells, a representative hepatocyte cell line derived from a hepatoblastoma, the in-cell repair assay was performed to determine the kinetics of repair of εA at each codon. Codons 246 and 247 were chosen as known nonmutagenic sites, whereas codons 249, 179 and 255 were selected as known mutagenic sites for comparative repair studies. The εA-containing phagemids (246εA, 247εA, 249εA, 179εA and 255εA) were transfected into HepG2 cells, harvested and assayed in the same manner as described for the MEFs (Figure 3A). The *t*_1/2_ for 246εA and 247εA was 3.0 ± 0.01 and 16.5 h ± 0.7, respectively while *t*_1/2_ for 249εA, 179εA and 255εA ranged from 18.5 ± 0.7 to 20.5 h ± 0.7 (Figure 3B). Similarly, 50% of εA at codons 246 and 247 was repaired in human umbilical vein endothelial cells (HUVECs) at 4.0 ± 1.4 and 5.0 h ± 0.01, respectively while the *t*_1/2_ for εA at codons 249, 179 and 255 ranged from 9.1 ± 0.1 to 15.3 h ± 4.6 (Figure 4A and B). These data demonstrate that εA is repaired at a significantly slower rate at mutagenic codons compared to nonmutagenic codons in live human cells (*P*-value < 0.05, Figures 3B and 4B).

### The base excision step is rate-limiting and is responsible for differential repair rates of εA at mutation hotspots vs. non-hotspots

**Figure 5.**
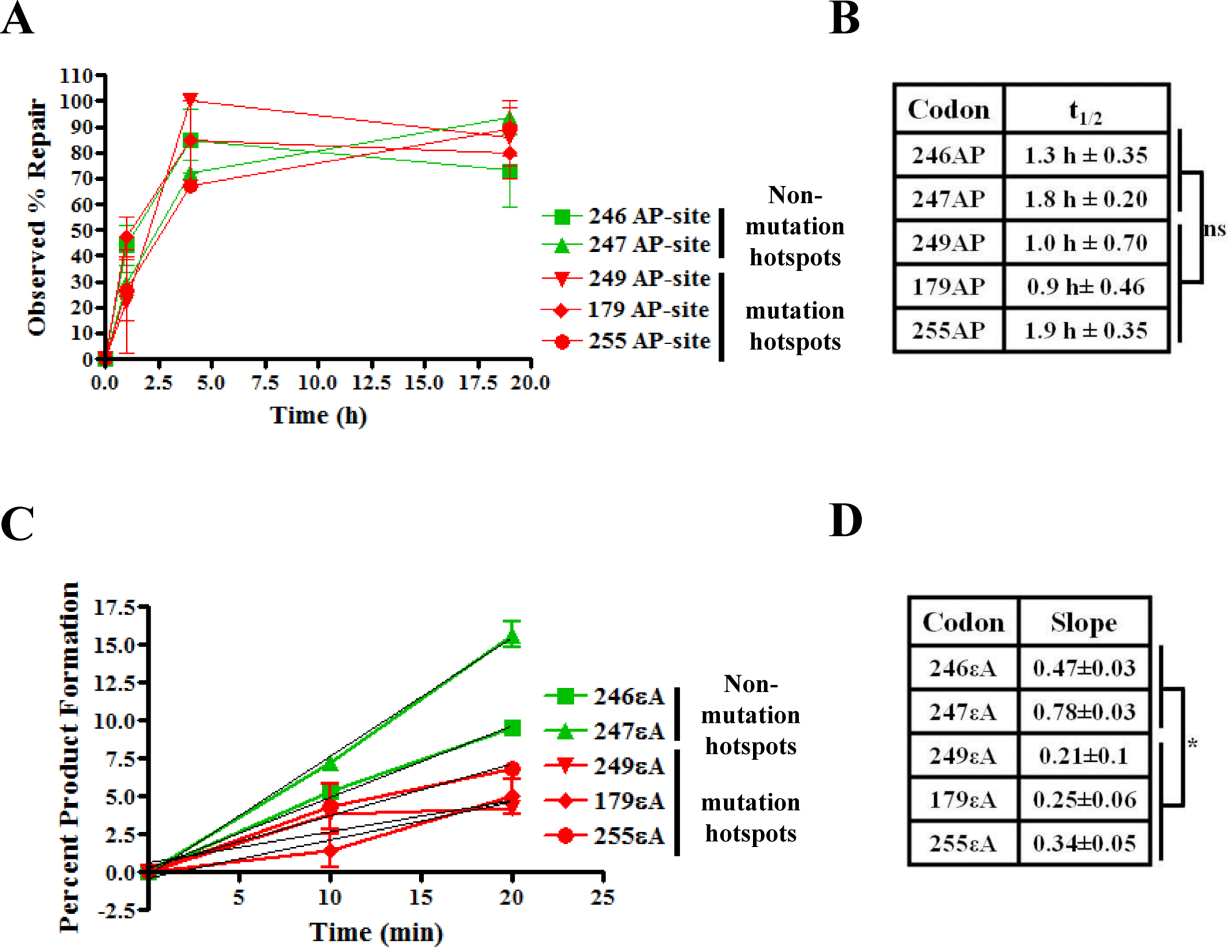
Role of base excision step in slow repair of εA at mutation hotspots. (**A**) AP-site repair in HepG2 cells. For each graphed point, *N* = 3, and error bars denote SEM. (**B**) Table reports *t*_1/2_, indicating the length of time required for 50% repair of AP-site. ns, not significant, *P*-value > 0.05, unpaired two-tailed Student's *t*-test comparing all non-hotspot *t*_1/2_ values with all hotspot *t*_1/2_ values. (**C**) MPG excision activity assay in HepG2 nuclear extracts. Graph indicates the percent product formation. Green denotes non-mutation hotspot codons and red denotes mutation hotspots. (**D**) Table reports the slope of the trendline for product formation over time. *P-value < 0.05, unpaired two-tailed Student's *t*-test comparing all non-hotspot *t*_1/2_ values with all hotspot *t*_1/2_ values.

While MPG initiates repair of εA by excising the damaged base and may have sequence-specific repair efficiency, the subsequent enzymes in the BER pathway may also have sequence-specific efficiency. In order to determine whether the slower rate of complete repair of εA at mutation hotspots was due to base excision or post-base excision steps, M13mp18-p53 phagemids with a single abasic (AP) site, which is the product of base excision and substrate for the next enzyme APE1, at the codons of interest were transfected into HepG2 cells and assayed for complete repair (Figure 5A). The εA-M13mp18-p53 phagemid DNA was treated with excess MPG to generate AP-M13mp18-p53 DNA and subsequently transfected into human cells for the repair assay (see ‘Materials and Methods’ section in the Supporting Information for details). An aliquot of MPG-treated phagemid DNA was reacted with APE1 and resolved on a 1% agarose gel to confirm the presence of AP-sites (Supplementary Figure S2, ≈95% reacted with MPG and APE1). It is known that the natural AP-sites used in this experiment are highly labile and may be converted to single strand breaks readily, possibly prior to transfection. However, the incision of the AP-site has been shown to be extremely efficient and is not the rate-limiting step of BER (33). Therefore, we considered gap-filling and ligation to be the most probable rate-limiting post-excision steps and did not consider the contribution of spuriously incised AP-sites to the results. No significant difference in *t*_1/2_ of the AP-site repair at various codons was observed (median *t*_1/2_ for non-hotspots and hotspots = 1.5 and 1.3 h, respectively) indicating that there was no significant difference in repair of AP-sites at the mutation hotspot and non-hotspot codons (*P*-value > 0.05, Figure 5B).

**Figure 6.**
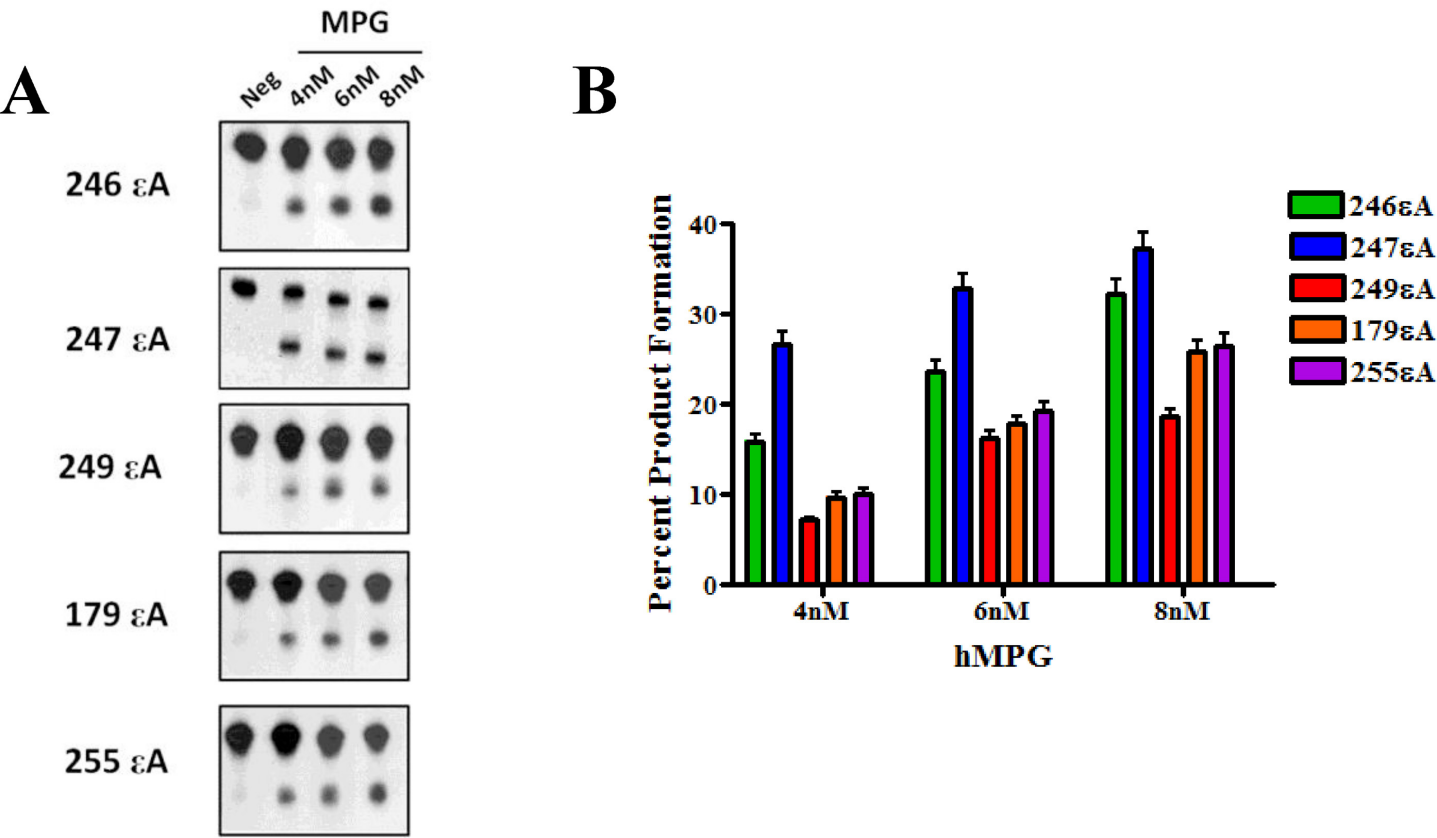
Reduced MPG excision activity on εA at mutation hotspots *in vitro*. (**A**) *In vitro* MPG activity assay using purified MPG and sequence context-specific εA substrate oligonucleotides. (**B**) Quantification of *in vitro* MPG activity assay.

Since the base excision steps are governed by MPG, nuclear extracts from HepG2 were assayed for MPG activity over time (Figure 5C and D) using five 50-mer oligonucleotides containing εA in the various sequence contexts. Ethenoadenine at non-hotspot codons was excised more efficiently than at mutation hotspots, which was quantified by the slope of the trendline of percentage product formation over time (Figure 5D). The slopes of the trendlines for the non-hotspots were significantly greater than the slopes of the trendlines for the hotspots (*P*-value < 0.05, Figure 5D). Similar results were observed using purified MPG in the activity assay. Compared to codons 246 and 247 MPG produced 2- and 2.5-fold less product, respectively, when εA was placed at codon 249 and 1.3- and 1.5-fold less product, respectively, when εA was placed at codon 179 or 255 (Figure 6A and B). The results from these experiments indicate that base excision steps, which are governed by MPG, are responsible for the slower rate of repair of εA at mutation sites. In fact, the *in vitro* MPG activity experiments with both extracts and purified protein consistently reported a 2–2.5-fold increase in MPG activity at non-mutation hotspot sequences over mutation hotspot sequences. These differences were consistent with the fold change in *t*_1/2_ values observed in the in-cell repair experiments, which also reported a 2–3-fold increase in MPG activity at non-hotspot codons versus hotspot codons (Figures 3 and 4).

### Slow turnover of MPG is the mechanism of slow repair of εA at mutation hotspots

Since base excision steps were responsible for the slower rate of repair of εA at mutation hotspots in p53, a comprehensive mechanism analysis was performed to determine which step in the excision reaction accounted for the differential rates in repair at mutation hotspot versus non-mutation hotspot sequences. Three intermediate steps in the excision reaction were tested: binding (equilibrium dissociation constant, *K*_D_), catalysis (*k*_chem_) and product dissociation (*k*_pd_), according to Michealis–Menten kinetics.

\begin{equation*} {\rm E} + {\rm S}\mathop\rightleftharpoons\limits^{K_{\rm D} } {\rm ES}\mathop{\begin{array}{l}\\-\!\!\!\rightharpoonup\\ \leftharpoondown\end{array}}\limits^{k_{{\rm chem}} } {\rm EP}\mathop\rightleftharpoons\limits^{k_{{\rm pd}} } {\rm E} + {\rm P} \end{equation*}

**Figure 7.**
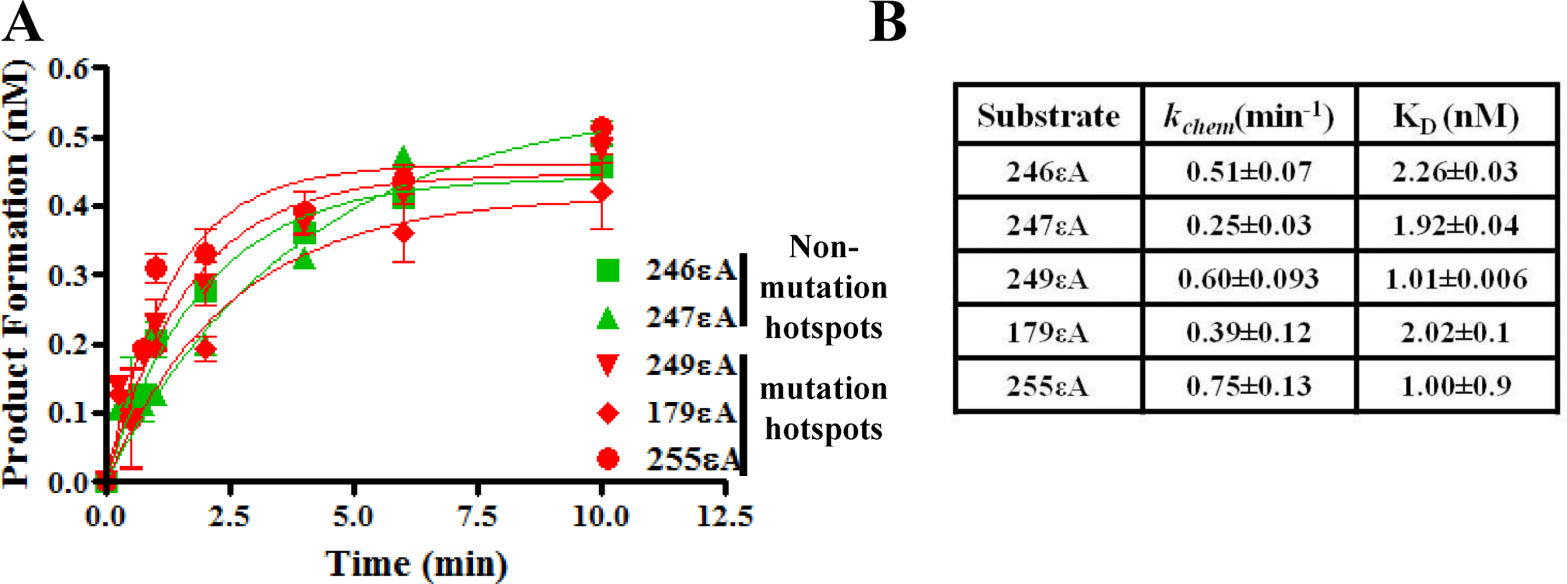
Effect of sequence context on kinetics of εA binding and catalysis by MPG at mutation hotspot codons. (**A**) Effect of sequence context on the catalysis rate of MPG. Green denotes non-mutation hotspot codons and red denotes mutation hotspots. For each graphed point, *N* = 3, and error bars denote SEM. (**B**) Table reports *k*_chem_ data from panel (A), which was derived from the equation described in the ‘Materials and Methods’ section ‘STO kinetic study’. The second column of the table reports the *K*_D_ of MPG for each of the oligonucleotide substrates, which was determined from SPR studies. The SPR methods and analysis are described in the ‘Materials and Methods’ section, and the Langmuir isotherms are reported in the Supporting Information, Figure S3.

**Figure 8.**
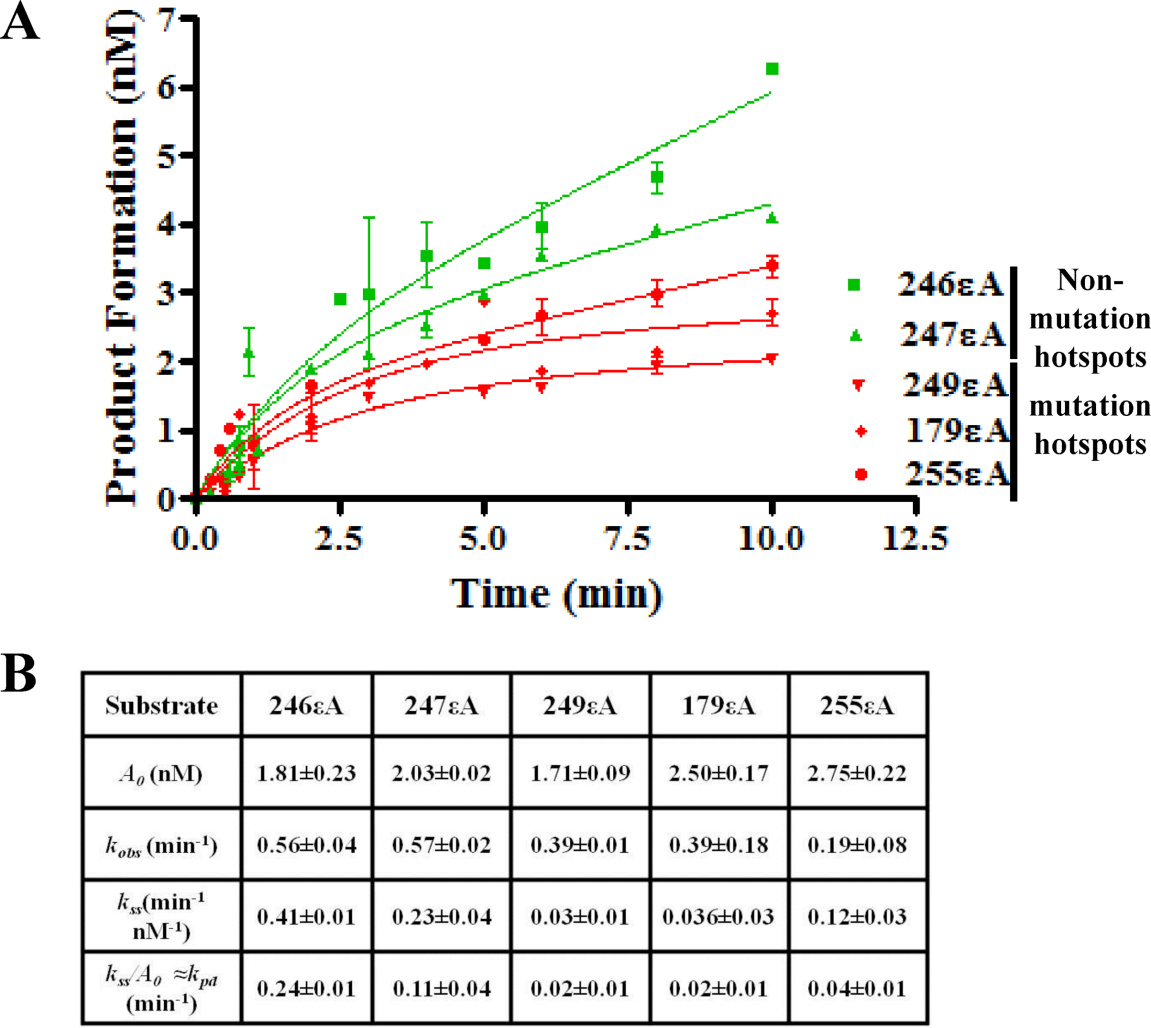
Effect of sequence context on product dissociation of MPG using εA-containing oligonucleotides. (**A**) Effect of εA sequence context on the product dissociation rate of MPG. Green denotes non-mutation hotspot codons and red denotes mutation hotspots. For each graphed point, *N* = 3, and error bars denote SEM. (**B**) Table reports the results of the analysis of panel (A), using the equation described in the ‘Materials and Methods’ section ‘Burst analysis’. *A*_0_, active MPG concentration; *k*_obs_, burst constant; *k_ss_/A*_0_ (≈*k*_pd_), product dissociation rate constant or turnover number.

The analysis included Surface Plasmon Resonance (SPR) spectroscopy to measure the DNA binding of MPG toward εA in different sequence contexts. Also, intermediate reaction steps were tested by pre-steady-state kinetic analysis. The sequence context was tested for its effect on the glycosidic bond cleavage step by STO kinetics and on the product dissociation step by multiple-turnover (MTO) kinetics. No difference in *k*_chem_ or *K*_D_ was observed among the five sequences in the STO and SPR studies, respectively, indicating that the sequence context did not affect the affinity of MPG for εA or its rate of glycosidic bond cleavage (Figure 7A and B and Supplementary Figure S3). On the other hand, a dramatic difference in product dissociation rate was observed among the five sequences in the MTO analysis. The *k*_pd_ for MPG on codons 246 and 247 was 0.24 ± 0.01 and 0.11 min^-1^ ± 0.04, respectively, while *k*_pd_ for MPG on the mutation hotspot substrates (0.02 ± 0.01 to 0.04 min^-1^± 0.01) was 3–12-fold lower (Figure 8A and B). The data presented in Supplementary Figure S4A are the MPG active site titration results using 246εA as substrate and demonstrate that the preparation of MPG used for the εA MTO analysis was 22% active (average of three determinations). Of note, the *k*_pd_ determined for MPG at the 246 sequence was comparable to the *k*_pd_ of MPG for εA in a random sequence context (0.26 min^-1^± 0.03), as determined previously using another preparation of MPG with an active concentration of ≈71% (28). Additionally, we tested the 22% active preparation of MPG used in the present εA study on the random εA sequence used in the previous publication and observed a similar *k*_pd_ value (data not shown) (28). The results of the mechanism analysis indicate that the slower overall rate of repair of εA at mutation hotspots is due to low MPG turnover, suggesting one of the potential mechanisms for the presence and pattern of mutation hotspots in p53 in inflammation- and VC-induced malignancies.

**Figure 9.**
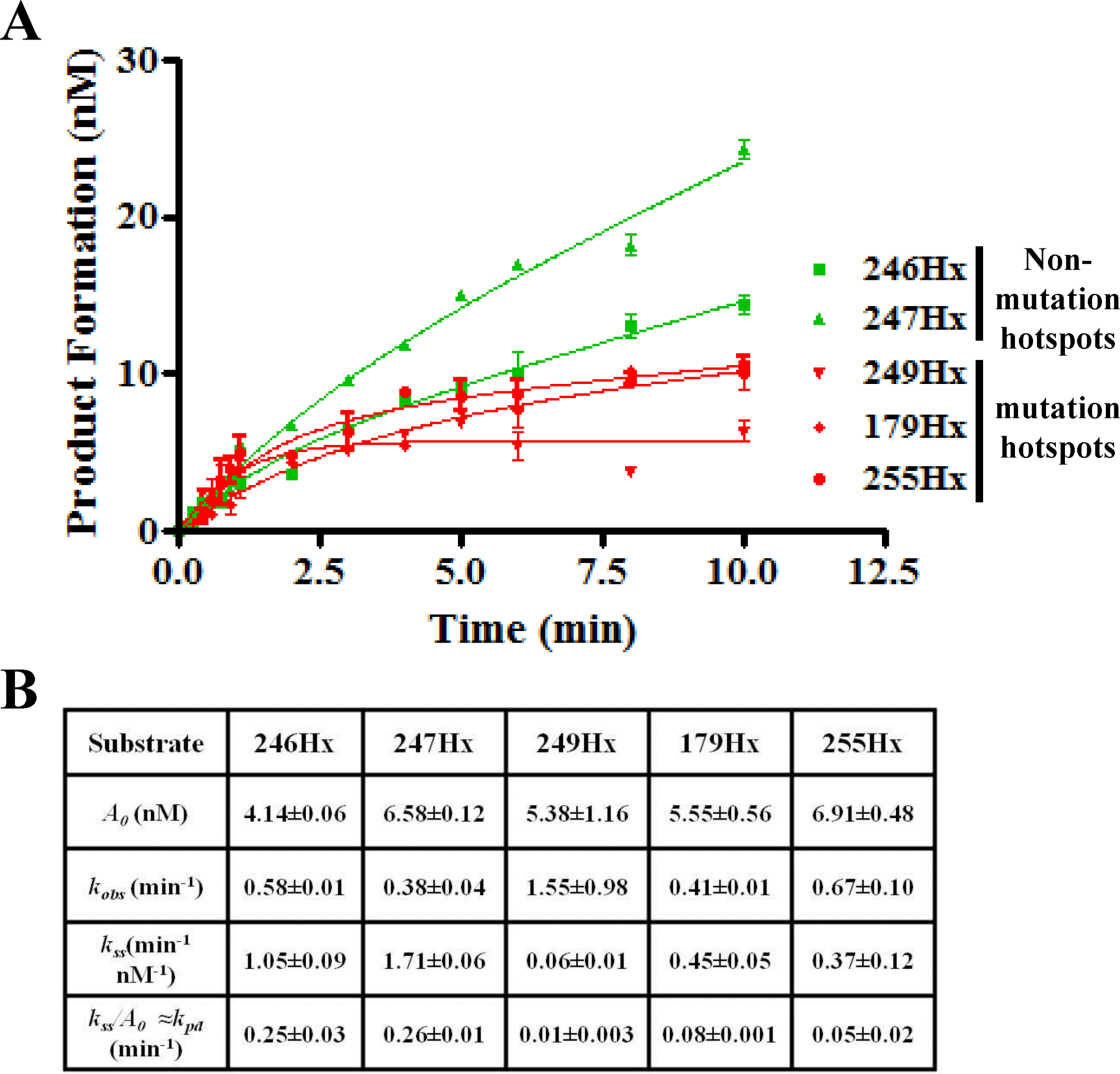
Effect of sequence context on product dissociation of MPG using Hx-containing oligonucleotides. (**A**) Effect of Hx sequence context on the product dissociation rate of MPG. Green denotes non-mutation sites and red denotes mutation sites. For each graphed point, *N* = 3, and error bars denote SEM. (**B**) Table reports the results of the analysis of panel (A), using the equation described in the ‘Materials and Methods’ section ‘Burst analysis’. *A*_0_, active MPG concentration; *k*_obs_, burst constant; *k_ss_/A*_0_ (≈*k*_pd_), product dissociation rate constant or turnover number.

Notably, the sequence-context of the product of MPG excision, the AP-site, is determining the rate of the excision reaction. Therefore, the turnover of another substrate of MPG, hypoxanthine (Hx), was tested in the individual codon contexts since the AP-site product is the same regardless of the adduct excised. The *k*_pd_ for MPG at the non-mutation sites was 0.25 ± 0.03 and 0.26 min^-1^± 0.01 for 246Hx and 247Hx, respectively (Figure 9). At mutation sites the *k*_pd_ determined for MPG was 3–26-fold slower (0.01 ± 0.003 to 0.08 min^-1^± 0.001), which was consistent with the pattern we observed for MPG and εA at the various codons (Figure 9). The MTO analysis of Hx-containing oligonucleotides was performed with a new preparation of purified MPG. The data presented in Supplementary Figure S4B are the MPG active site titration results using 246Hx as substrate and demonstrate that the new preparation of MPG used for the Hx MTO analysis was 83% active (average of three determinations).

## DISCUSSION

Our study revealed that slow repair of εA at mutation hotspots in p53 was caused by sequence-specific low turnover of the repair-initiating glycosylase, MPG. The present work has revealed several important points regarding sequence-specific DNA repair. This study is the first to demonstrate codon-specific delayed repair of a DNA adduct in live human cells in a biologically relevant sequence by modification of a previously described method (34). Notably, the codon-specific deficiency was at mutation hotspot sequences in the p53 gene. Furthermore, the enzymatic mechanism for slower sequence-specific repair was demonstrated by a comprehensive study of the repair-initiating excision step both in-cell and *in vitro*. The observed slow repair of εA implicated MPG-initiated BER as the primary pathway responsible for the difference in repair efficiency. The BER pathway includes several enzymes working in a sequential manner. In the case of MPG-initiated BER, MPG recognizes the adduct (εA) and cleaves the glycosidic bond between the damaged base and the sugar, leaving an AP-site. The AP-site is subsequently cleaved by APE1 resulting in a nick in the DNA strand, where DNA polymerase β (Polβ) incorporates a nucleotide and DNA ligase III (LigIII) seals the nick in the phosphate-sugar backbone, resulting in the fully restored sequence (35). Importantly, since there was no significant difference in the repair of AP-sites between non-hotspot and hotspot codons (Figure 5A), we conclude here that post-base excision step(s) do not contribute to the slower repair of εA at mutation hotspots. Our mechanistic studies, therefore, focused on characterizing the interaction of MPG, the base excising glycosylase, with εA in different sequence contexts. Using the *in vitro* assay, we showed with both cell-free extracts and purified enzyme that MPG exhibits less activity toward εA placed at mutation hotspot codons than non-hotspot codons, which was consistent with the results for complete repair (εA to A) we observed in cells (Figures 3, 4, 5C, D and 6). Moreover, our activity experiments in nuclear extracts over different time points correlate well with the observed excision rate differences in the burst analysis (Figures 5D and 8).

Previously, we and others determined that the excised damaged base, N-terminal tail of MPG, Mg^2+^ and even other BER proteins, such as APE1 and proliferating cell nuclear antigen (PCNA) can regulate the turnover of MPG but in this study we have also demonstrated the importance of sequence context in the rate of product dissociation for MPG (Figures 8 and 9) (21,28,36–38). In this case, AP-site DNA is the product that confers sequence-specificity, since the excised base, εA, is the same for all sequences. Base flipping by MPG, possibly facilitated by sequence context, is part of εA recognition and subsequent catalysis but is not associated with product (AP-site) binding and inhibition of MPG as the εA base is excised. Burst analysis with Hx in the various sequence contexts confirmed that the AP-site product is playing a role in determining codon-specific turnover (Figure 9). It should be noted, however, that the pattern of *k*_pd_ values observed for the Hx burst analysis was slightly different than that of the εA burst analysis, which indicates that the excised base, acting as a second product, is playing some role in the codon-specific turnover. In future studies, it would be useful to examine the structure of AP-site- and excised base-bound MPG in the mutation hotspot and non-hotspot sequence contexts, as such a study would provide valuable insight into the mechanism of sequence-specific turnover rate. However, as previously mentioned, we have shown that the N-terminal tail is required for the turnover of MPG, indicating the role of this motif in product dissociation (37). Currently, the crystal structure of the full length MPG has not been solved, rather the solved structure is a truncated form without the N-terminal tail (39). This study highlights the need for a full length MPG crystal structure in order to correctly carry out a sequence-specific structure–function analysis of the product dissociation by MPG.

The sequence context-specific turnover rate observed in this study has profound biological significance since it was at mutation hotspot codons that MPG exhibited slow rates of product dissociation. Overall εA may persist at mutation hotspot sequences because of the slow rate of turnover by MPG, increasing both the chance of erroneous base incorporation opposite the damaged base during DNA replication and, consequently, the mutation frequency at those sites. Ethenoadenine is a strong replication blocker although its bypass during translesion synthesis (TLS) has been shown to be erroneous but relatively efficient by human DNA polymerase η (Polη) compared to other TLS polymerases (40–42). The results of the referred studies demonstrate the genotoxicity of εA both *in vitro* and *in vivo*, implicating the importance of repair of this lesion in the prevention of mutagenesis. Understanding both the repair and genotoxicity of this lesion is particularly important since εA is formed by lipid peroxidation products, which are present at high levels in a variety of inflammatory conditions. Levels of εA in human cells have been correlated with risk for various types of oxidative stress-associated cancers (43,44). The present study also has implications for mutation patterns associated with other mutagenic adduct substrates of MPG since it excises a variety of adducts, including alkylated, deaminated and lipid peroxidation-induced purine adducts (45–47).

The activity of BER glycosylases has been shown to prevent intestinal and lung carcinogenesis in mice, with or without treatment with a damaging agent, as well as humans (48–50). Specifically, the enzyme of interest in the present study, MPG, has been shown to be important in preventing cancer in a mouse model of inflammation-mediated colon carcinogenesis (51). As these referred studies highlight the significance of BER glycosylase activity in cancer prevention, it is highly significant that we observed sequence-specific excision deficiency in a normal, wild-type glycosylase. The local sequence-directed impedance of excision activity may be playing a role in spontaneous carcinogenesis, potentially unrelated to any inherited defect in the glycosylase. Interestingly, single nucleotide polymorphisms (SNPs) of MPG have been identified in the human population, so it remains to be seen if these alterations could lead to modifications in the observed sequence-specific turnover of the wild-type enzyme, which may re-direct mutation signatures in a SNP-specific manner (52).

It should be noted that previous studies have examined mutation hotspots as a marker for unrepaired lesions, resulting from either inefficient repair of adducts or excess adduct formation by genotoxic agents. Ligation-mediated PCR (LM-PCR) is a powerful tool that has been utilized in several studies as a method of monitoring the location of DNA lesions induced by various damaging agents. Using LM-PCR it was found that benzo(a)pyrene diolepoxide (BPDE), a metabolite of benzo(a)pyrene, present in cigarettes, preferentially formed adducts at known human lung cancer mutation hotspots in p53 (53). This observation suggested sequence-specificity of the genotoxic reaction and provided a mechanism for high frequency of mutation at certain sites. However, in addition to the rate of lesion formation, the rate of repair of DNA adducts is an equally important component of the mutagenic outcome of an adduct. In fact, erroneous base incorporation opposite lesions in ssDNA is drastically higher than that in duplex DNA, presumably because of lack of efficient repair in the context of ssDNA, underscoring the contribution of repair in overall mutation frequency (41). The rate of damage repair was examined in another LM-PCR-based study with UV-irradiated human skin fibroblasts. Analysis of the location and persistence of UV-induced DNA damage in the p53 gene indicated slower removal of damage at known human skin cancer mutation hotspots, suggesting that sequence-specific deficiency in repair was responsible for the persistence of mutagenic adducts at specific sites, making those mutation hotspots (54). However, it was not clear whether the disappearance of DNA damage was due to repair or dilution of the adduct level by DNA replication. Moreover, even if the damage disappearance over time is defined as repair, the mechanism of sequence-specific differences in removal of DNA adducts was not studied. Our present study not only focused on comparing in-cell repair rates of an adduct in a sequence-specific manner, but also on establishing a mechanism of differential repair efficiency. Additionally, while the LM-PCR method was valuable because of its ability to establish the presence and exact location of adducts, another major limitation is the uncertainty about the specific type of damage being observed. The strength of the method developed and used in our study is that a known DNA adduct is specifically engineered into a non-replicating plasmid and assayed for repair. More importantly, no damaging agent treatment is required for our assay, so the observed repair rates represent the basal condition of the cells rather than a treatment-induced condition, which may obscure the normal behavior of the repair pathways.

It is true that the present study uses a surrogate system to monitor repair in live cells rather than direct monitoring of repair in genomic DNA (gDNA). One concern may be that this plasmid DNA-based assay may not provide insight into biologically relevant DNA–protein interactions within chromatinized nuclear DNA. However, reporter plasmid DNA has been known to form a chromatin-like structure in cells and reliably mimic nuclear DNA structure (55,56). In fact, promoter activity is often measured using the luciferase-based reporter assay, which is the gold standard method used extensively in gene expression and transcription mechanism studies and relies on a plasmid-based system to understand nuclear biology.

In conclusion, we have observed significantly delayed repair of a lipid peroxidation-induced DNA adduct at known mutation hotspots in p53 in cells and established for the first time that the mechanism of differential repair was glycosylase turnover rate. It is true that DNA–protein interactions in cells are complex, and we cannot exclude the contribution of some as yet unknown factors to overall slower repair of εA at mutation hotspot codons. We also acknowledge that mutations at these codons confer a selective advantage to cancer cells, and this advantage may also play a significant role in the mutation hotspot pattern in p53 observed in human cancer cells. However, the results of our present study and the work of others demonstrate the impact of DNA repair on mutation pattern and carcinogenesis. We posit that the mechanism of slow repair presented here could have broad relevance, possibly as the architect of hotspot signatures in other cancer-related genes in addition to p53 and in other cancer types as well.

## SUPPLEMENTARY DATA

Supplementary Data are available at NAR Online.

SUPPLEMENTARY DATA
