# Supplementary material for: Slow repair of lipid peroxidation-induced DNA damage at p53 mutation hotspots in human cells caused by low turnover of a DNA glycosylase
Source: Nucleic Acids Res. 2014 Jul 31;42(14):9033–46. doi: 10.1093/nar/gku520 (PMC4132702; doi:10.1093/nar/gku520)
Supplement: SUPPLEMENTARY DATA [file supp_42_14_9033__index.html]

Slow repair of lipid peroxidation-induced DNA damage at p53 mutation hotspots in human cells caused by low turnover of a DNA glycosylase — SUPPLEMENTARY DATA 

# Slow repair of lipid peroxidation-induced DNA damage at p53 mutation hotspots in human cells caused by low turnover of a DNA glycosylase

## SUPPLEMENTARY DATA

**Files in this Data Supplement:**

- SUPPLEMENTARY DATA
