# Supplementary material for: Slow repair of lipid peroxidation-induced DNA damage at p53 mutation hotspots in human cells caused by low turnover of a DNA glycosylase
Source: Nucleic Acids Res. 2014 Jul 31;42(14):9033–46. doi: 10.1093/nar/gku520 (PMC4132702; doi:10.1093/nar/gku520)
Supplement: SUPPLEMENTARY DATA [file supp_gku520_nar-01198-d-2014-File011.pdf]

Supplementary Information

Materials and Methods

εA-M13mp18-p53 and APsite-M13mp18-p53 in vitro construct preparation

Typically, for each preparation of εA-M13mp18-p53, 6 individual reactions were performed simultaneously according to the following protocol. Phosphorylation of the primers was performed by incubating 2 µg of codon-specific εA-containing oligonucleotide (2 µL) with 1X PNK buffer, 400 nM ATP, 50 mM DTT, and 10 U of T4 polunucleotide kinase (New England Biolabs, Ipswich, MA) in a 30 µL reaction volume at 37°C for 45 mins. The phosphorylated oligonucleotide was purified through a G-25 column (GE Healthcare Life Sciences, Pittsburgh, PA) according to the manufacturer's protocol. Then 6 µL of this purified oligonucleotide was incubated with 2 µg of p53-M13mp18 ssDNA in an annealing buffer containing 10 mM Tris-HCl, pH 7.5, and 50 mM NaCl in a 20 µL reaction volume. This annealing reaction was incubated at 80°C for 5 minutes and slowly cooled to room temperature with brief centrifugation when the reaction reached 50°C. Then the annealing reaction was incubated with an extension reaction mixture containing 1X T7 DNA polymerase buffer, 1.5 mM ATP,1.5 mM of each dNTP, 10 mM DTT, and 160 µg/mL BSA, 10 U of T7 DNA polymerase (New England Biolabs, Ipswich, MA), and 400 U of T4 DNA ligase (New England Biolabs, Ipswich, MA) in a final reaction volume of 30 µL for 5 mins on ice followed by 5 mins at room temperature. The extension reaction was subsequently incubated at 37°C for 1 h. After an hour, 50 nmol of ATP and 200 U of T4 DNA ligase was added to the extension reaction and incubated at 14°C overnight for efficient ligation to occur. The 6 individual reactions were then pooled together and incubated with 1X Supercoil-It buffer (Bayou Biolabs, Metairie, LA) and 2 µL of Supercoil-It enzyme mixture at 37°C for 3 h. Plasmid was recovered after the incubation by purification using Qiaquick PCR Purification kit (Qiagen, Gaithersburg, MD). The DNA was eluted from the column using 50 µL of molecular grade water. Concentration of the eluted DNA was measured using a Nanodrop spectrophotometer, and the DNA was stored at -20°C.

APsite-M13mp18-p53 was prepared by incubating 1pmol εA-M13mp18-p53 phagemid with 100 µM MPG (expressed and purified previously, (36)) in 200 µL of reaction buffer containing 50 mM Tris-HCl, pH 8.0, 50 mM KCl, 0.5 mM DTT, and 500 µg/mL BSA at 37°C for 45 mins. Then another 20 pmol of MPG was added to the reaction and incubated for another 45 mins at 37°C. An aliquot was digested with 25 nM APE (expressed and purified previously,(57)) and 10 mM $MgCl_2$ and resolved on an agarose gel to confirm AP site generation (*Figure S2*). APsite-M13mp18-p53 DNA was immediately transfected into HepG2 cells as described for εA-M13mp18-p53 DNA. *Figure S2* also demonstrates the quality of the prepared construct (see "untreated" lane). Approximately 95% of εA-M13mp18-p53 DNA was converted to nicked DNA in the presence of MPG and APE (see "MPG + APE" lane). We predict that all εA-M13mp18-p53 plasmid molecules contain εA, and that the remaining unreacted 5% of molecules represent a limitation of MPG activity in the reaction.

In vivo repair of εA and AP-sites in human cells

Mixing Experiment: Eight hundred picograms (pg) control (undamaged) M13mp18-p53 phagemids and plasmids containing a single εA at codon 246, 247, 249, 179, or 255 were digested with HindIII and XbaI (see description of restriction digestions below). The digested control M13mp18-p53 plasmid was then mixed with εA M13mp18-p53 plasmid to create the following percentages of εA-containing DNA: 0, 10, 30, 50, 70, 90, and 100%. The mixed ratios were subsequently processed as described in the following sections.

Transfection and retrieval of episomal DNA: Approximately 300,000 HepG2 cells or HUVECs were transfected with 1µg control, εA, or AP-site M13mp18-p53 plasmid using Lipofectamine 2000 (Life Technologies, Grand Island, NY)i in Opti-MEM media (Life Technologies, Grand Island, NY) according to the manufacturer's protocol in 6-well plates. Cells were then incubated at 37°C, and plasmid was retrieved at indicated times 5 to 48 h post-transfection for εA-containing plasmid, or 1.5-20 h post-transfection for AP-site-containing plasmid. To harvest transfected episomal DNA, cells were washed twice with 1X PBS, trypsinized, and pelleted. The pelleted cells were washed once with 1X PBS and again pelleted. The plasmid DNA was isolated from the cells using the Qiagen Spin Miniprep Kit (Qiagen, Gaithersburg, MD) according to manufacturer's protocol for bacterial pellets.

Single-stranded p53 probe preparation: To produce biotinylated, uracil-containing p53 probe, PCR reactions were carried out using control M13mp18-p53 DNA as template. The primer sequences are listed in S1. Each 50 μL reaction contained 1X GoTaq reaction buffer (Promega, Madison, WI), 200 nM each of dATP, dGTP, dCTP, and dUTP (Life Technologies, Grand Island, NY), 500 nM each of primer 1 and 2, 1ng template DNA, and 1U GoTaq polymerase (Promega, Madison, WI). PCR conditions were as follows: 3 mins at 95°C, 35 cycles of 15 s at 95°C, 30 s at 60°C, and 1.5 min at 72°C, followed by a 5 min final extension at 72°C. PCR products were purified using Qiagen PCR purification kit (Qiagen, Gaithersburg, MD) according to manufacturer's instructions. The eluted purified biotinylated p53 PCR product was subsequently immobilized on streptavidin-coated magnetic beads using the Dynabeads® kilobase Binder Kit (Life Technologies, Grand Island, NY) according to manufacturer's protocol with some modifications. Prior to incubation with the biotinylated PCR product, the magnetic beads were pre-incubated in the provided binding buffer to minimize non-specific binding of DNA to the beads. After 1h of immobilization at room temperature (according to the given protocol), the tubes were placed on a magnetic separator, and the supernatant was removed. The beads were washed twice with the provided wash buffer and once with molecular grade water. The beads were then resuspended in 50 μL 10 mM Tris-HCl, pH 7.5. The solution was incubated at 100°C for 5 mins then immediately placed on the magnetic separator and the supernatant removed and discarded. The single-stranded p53 probe, immobilized on the remaining magnetic beads, was resuspended in 100 μL 1 M NaCl and subsequently hybridized to HindIII/XbaI-digested M13mp18-p53 DNA.

Restriction Digestion of Plasmids: Retrieved plasmid DNA post-transfection was concentrated to approximately 5 μL. Each 20 μL digestion reaction contained 1X NEB Buffer 2, 100 μg/mL BSA, 20 U HindIII (New England Biolabs, Ipswich, MA), 20 U XbaI (New England Biolabs, Ipswich, MA), and the concentrated retrieved plasmid DNA. Reactions were incubated at 37°C for 2 h and subsequently diluted 25 times in molecular grade water before hybridization to single-stranded p53 probe.

Hybridization: Hybridization reactions containing 10 μL single-stranded p53 probe and 10 μL diluted HindIII/XbaI digested plasmid were incubated at the following temperatures in an Eppendorf Mastercycler machine: 5 min at 95°C, then decreasing 1°C every 15 min to 60°C, 30 min at 60°C, and finally decreasing to room temperature at 1°C per minute. For the mixing experiment, each hybridization reaction contained 10 μL single-stranded p53 probe and 10 μL (400pg) mixed HindIII/XbaI-digested M13mp18-p53 DNA.

MPG/APE/UNG digestion: Hybridized samples were placed on separation magnet (Perkin Elmer, Waltham, MA), and the supernatant was removed. The beads were resuspended in a 50 μL reaction mixture containing 50 mM Tris-HCl, pH 8.0, 50 mM KCl, 0.5 mM DTT, 2 mg/mL BSA, and 24 nM MPG (expressed and purified previously,36). The MPG reactions were incubated at 37°C for 30 min after which another 1nmol of MPG was added to the reaction. The reactions were incubated at 37°C for another 30 min, then 10 μL of an APE1/$MgCl_2$ mixture (75 nM APE1 with 50 mM $MgCl_2$) was added and the reactions incubated at 37°C for 15 min followed by 10 min at 70°C. For experiments with AP-site plasmid, the hybridized samples were treated with APE1/$MgCl_2$ mixture only. The tubes were placed on the separator magnet, and the supernatant was removed. The beads were washed twice in 50 μL of a wash buffer containing 50 mM Tris-HCl, pH 8.0, 50 mM KCl, and 1 mM DTT. After removing the supernatant in the second wash, the beads were resuspended in 20 μL of a reaction mixture containing 50 mM Tris-HCl, pH 8.0, 100 mM NaCl, 10 mM $MgCl_2$, 1 mM DTT, and 10 U UNG. The UNG reactions were incubated at 37°C for 1 h after which another 10U of UNG was added to the reaction and incubated for another hour at 37°C. The digested samples were finally diluted 8 times in molecular grade H_2O and stored at 4°C.

Real time PCR: In order to determine the percent repair of εA or AP-site, PCR analysis was performed for two regions in the plasmid, the εA region and an undamaged control region. Each εA region or control region real time PCR reaction contained 1X Maxima Sybr Green qPCR Master Mix (Thermo Scientific, Waltham, MA), 500 nM of each primer (see *Table S1* for forward and reverse primer sequences), and 2 μL of diluted MPG/APE/UNG- or APE/UNG-treated samples. Reactions were carried out in a 96 well plate in a BioRad iCycler (BioRad, Hercules, CA) under the following cycling conditions: 10 min at 95°C followed by 40 cycles of 10 s at 95°C, 30 s at 53°C, 30 s at 72°C. After PCR, the repair percentages were calculated as previously described, using a ratio of damage region/control region C_t values for each sample (33).

Nuclear Extract Preparation

Nuclear extract was prepared using buffers and protocol as previously described with some modification (58). Briefly, 10^6 cells (HepG2 or MEFs) were scraped from a 10 cm plate with 400 µL of buffer A and collected into a microcentrifuge tube. The cells were incubated on ice for 15 mins. Then 25 µL of 10% Nonidet NP-40 (US Biological, Salem, MA) was added, and the sample was vortexed. Nuclei were pelleted by centrifuging the samples at 1500 rpm for 1 min at 4°C. The supernatant was removed, and the nuclei were resuspended in approximately 50 µL of buffer C. The samples were rocked vigorously at 4°C for 20 mins and subsequently centrifuged at 14,000 rpm for 10 mins at 4°C. The supernatant was aliquoted and stored at -80°C.

Preparation of ^{32}P-labeled oligonucleotide substrates

Sequence-specific εA-containing oligonucleotides and their complementary oligonucleotides were purchased from Gene Link (Hawthorne, NY). For excision activity, single turnover, and multiple turnover (burst) experiments, the εA-containing strand was labeled at the 5' end using T4 PNK and γ^{32}P-ATP (Perkin Elmer, Waltham, MA) and annealed to complementary oligonucleotide to prepare ^{32}P-end-labeled duplex oligonucleotide substrate.

References

57. Adhikari, S., Manthena, P.V., Sajwan, K., Kota, K.K. and Roy, R. (2010) A unified method for purification of basic proteins. *Analytical biochemistry*, **400**, 203-206.
58. Matthias, P., Muller, M.M., Schreiber, E., Rusconi, S. and Schaffner, W. (1989) Eukaryotic expression vectors for the analysis of mutant proteins. *Nucleic acids research*, **17**, 6418.

Figure Legends

S1. Adenine mutations in p53 in human cancers. (A) Graph depicts the percentage of tumors sampled that harbored adenine mutations in the p53 gene. Cancer types with the highest percentage of samples with adenine mutations are shown. Numbers above each bar indicate the number of tumors sampled from each type. (B) Codon distribution of adenine mutations in the cancer types represented in panel A. Labeled data points indicate codons where A mutations were found in >2% of tumors sampled. Data from IARC TP53 Database, R17 (16).

S2. *In vitro* εA-M13mp18-p53 and APsite-M13mp18 construct preparation. εA-M13mp18-p53 plasmid DNA was treated with excess MPG to generate APsite-M13mp18-p53 DNA. To determine the efficiency of MPG excision, an aliquot of MPG-treated plasmid was treated with APE1 and resolved on a 1% agarose gel, which shows ≈95% conversion of CCC DNA to nicked DNA.

S3. Langmuir isotherms of MPG binding to sequence-specific biotinylated εA-containing oligonucleotide substrates (see Supplementary Table 1) using Biacore-T100. The results of the binding experiments and fitted curves are represented by colored and black lines, respectively, for (A) 246εA (B) 247εA (C) 249εA (D) 179εA and (E) 255εA. Various concentrations of MPG (3 injections each) were used to obtain binding kinetic parameters: 0, 0.315, 0.625, 1.25, 2.5, 5, 10, 20, and 40 nM.

S4. Active site titration. Kinetics of the reaction of MPG with εA- or Hx-containing oligonucleotide substrate. (A) A 22% active preparation (average of 3 enzyme concentrations tested) of purified MPG was used in increasing concentrations (7, 10, and 20 nM) with excess εA-containing oligonucleotide substrate (see Materials and Methods for details). (B) A 83% active preparation (average of 3 enzyme concentrations tested) of purified MPG was used in increasing concentrations (3.5, 7, and 10 nM) with excess Hx-containing oligonucleotide substrate (see Materials and Methods for details). Tables report the A_0 (amplitude of the burst or active

concentration of enzyme) and the percent active concentration (denoted as %) as calculated from the ratio of A_0 over initial enzyme concentration used (A_0/E_0).

Table S1

Table S1. Substrate oligonucleotides utilized for activity assays, pre-steady state and binding kinetics of hMPG.

Oligonucleotide Name	Experimental use	Sequence (5'→3')[b]
M13mp53-p53-ctrl	eA-M13mp18-p53 preparation	TCCTGCATGGGCGGCATGAACCGGAGG
M13mp53-p53-246εA	eA-M13mp18-p53 preparation	TCCTGCATGGGCGGCXTGAACCGGAGG
M13mp53-p53-247εA	eA-M13mp18-p53 preparation	ATGGGCGGCATGXACCGGAGGCCC
M13mp53-p53-249εA	eA-M13mp18-p53 preparation	GCATGGGCGGCATGAACCGGXGGCCCATCCTCACC
M13mp53-p53-179εA	eA-M13mp18-p53 preparation	GGCGCTGCCCCCACCXTGAGCGCTG
M13mp53-p53-255εA	eA-M13mp18-p53 preparation	CCATCCTCACCATCXTCACACTGG
Probe F	Single-stranded p53 probe preparation	GCTTGGTACCGAGCTCGGAT
Probe R	Single-stranded p53 probe preparation	*CATCATATCAGTCTGAGTCAGGCCCTTC
246/247 F	Real time PCR	GCATGGGCGGCATGAACC
246/247 R	Real time PCR	AAACACGCACCTCAAAGCTGTTCC
249 F	Real time PCR	CATGAACCGGAGGCCCAT
249 R	Real time PCR	AAACACGCACCTCAAAGCTGTTCC
179 F	Real time PCR	CATGAGCGCTGCTCAGATAGC
179 R	Real time PCR	CTGTCATCCAAATACTCCACACGC
255 F	Real time PCR	TCCTCACCATCATCACACTG
255 R	Real time PCR	TTGCGGAGATTCTCTTCCTCTGTG
Ctrl F	Real time PCR	TGAAGACCCAGGTCCAGATG
Ctrl R	Real time PCR	CAGACTTGGCTGTCCCAGAA
246εA/Hx	Substrate (*in vitro* mechanism studies)[a]	GTGTAACAGTTCCTGCATGGGCGGCXTGAACCGGAGGCCCATCCTCACCATC
247εA/Hx	Substrate (*in vitro* mechanism studies[a]	GTAACAGTTCCTGCATGGGCGGCATGXACCGGAGGCCCATCCTCACCATCATC
249εA/Hx	Substrate (*in vitro* mechanism studies) [a]	GTTCCTGCATGGGCGGCATGAACCGGXGGCCCATCCTCACCATCATCACAC
179εA/Hx	Substrate (*in vitro* mechanism studies) [a]	GAGGTTGTGAGGCGCTGCCCCCACCXTGAGCGCTGCTCAGATAGCGATGG
255εA/Hx	Substrate (*in vitro* mechanism studies) [a]	GAACCGGAGGCCCATCCTCACCATCXTCACACTGGAAGACTCCAGTGGTAATC

[a] For activity, pre-steady state, binding kinetics, and *in vitro* bypass studies, oligonucleotide sequences shown are the εA-containing strand. For those assays the substrates were duplexed with appropriate complementary oligonucleotides. For binding kinetics experiments the complementary oligonucleotides were biotinylated on the 3' ends for immobilization on the streptavidin-coated Biacore chips.
[b] X denotes εA or Hx.

Figure S1

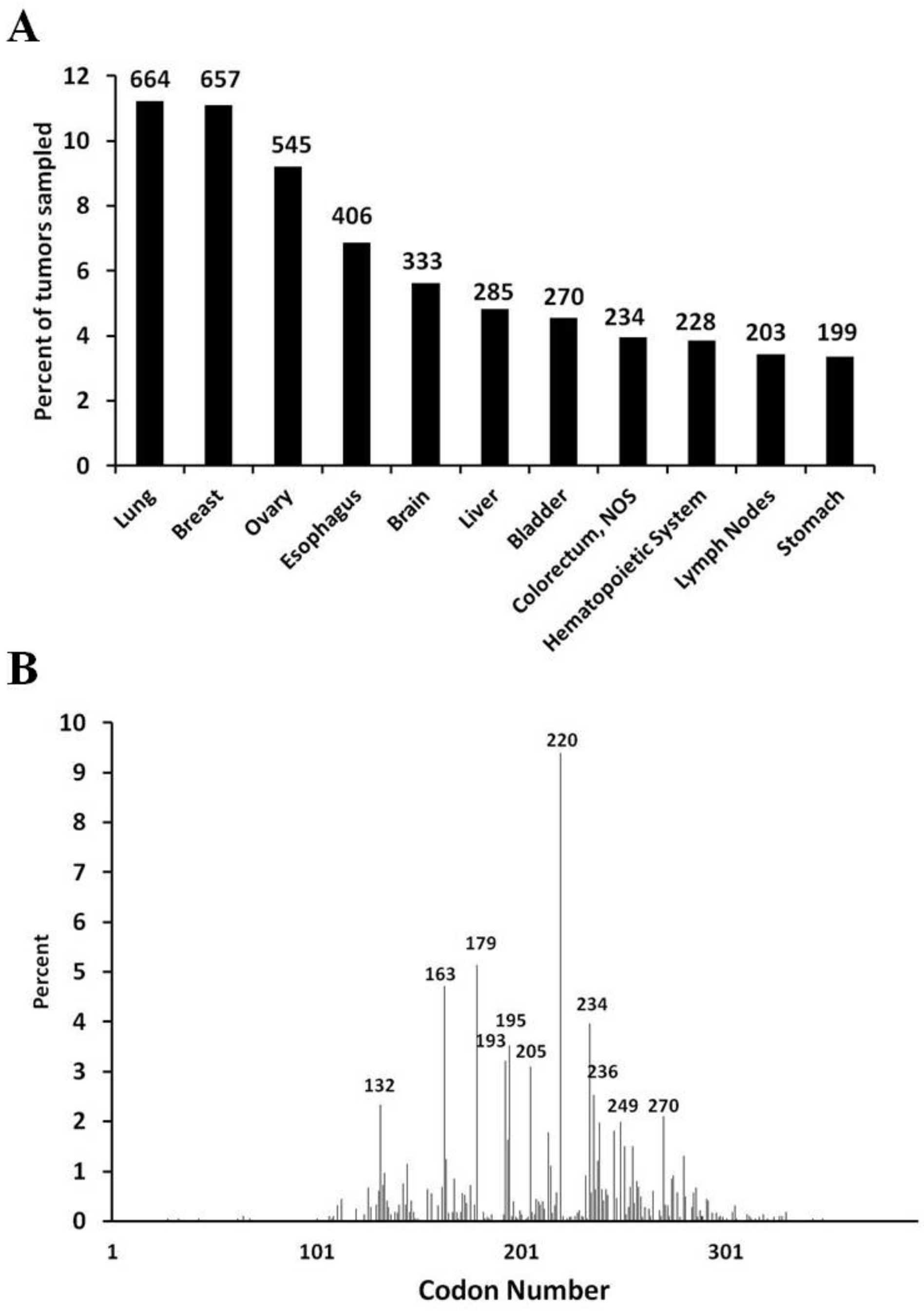
A
Percent of tumors sampled
664
657
545
406
333
285
270
234
228
203
199
Lung
Breast
Ovary
Esophagus
Brain
Liver
Bladder
Colorectum, NOS
Hematopoietic System
Lymph Nodes
Stomach
B
Percent
220
179
163
195
193
205
234
236
249
270
132
Codon Number

Figure S2

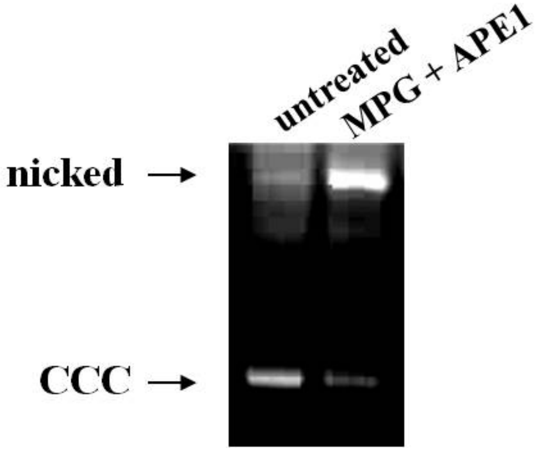

Figure S3

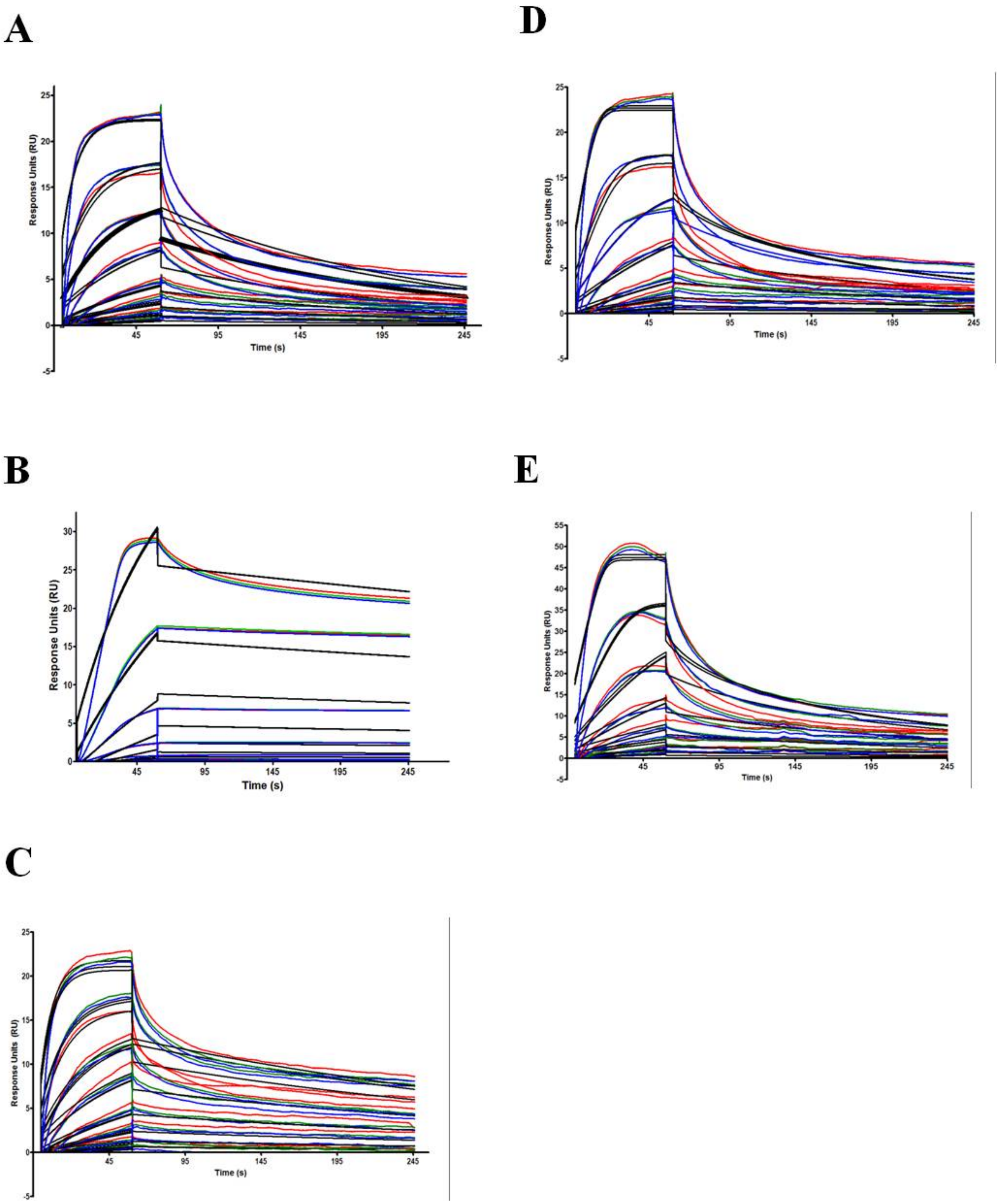

A
Response Units (RU)
Time (s)
B
Response Units (RU)
Time (s)
C
Response Units (RU)
Time (s)
D
Response Units (RU)
Time (s)
E
Response Units (RU)
Time (s)

Figure S4

A

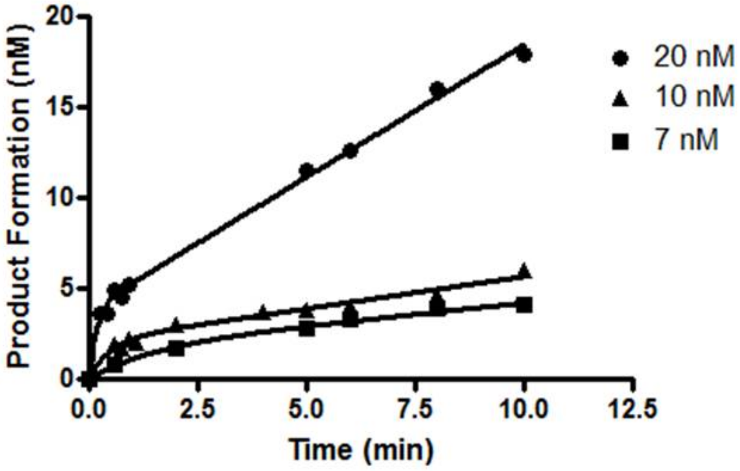

MPG* (εA)	A_0	%
**20 nM**	**3.9**	**19.5**
**10 nM**	**2.1**	**21.0**
**7 nM**	**1.7**	**24.3**

* Preparation of purified MPG used for all εA experiments

B

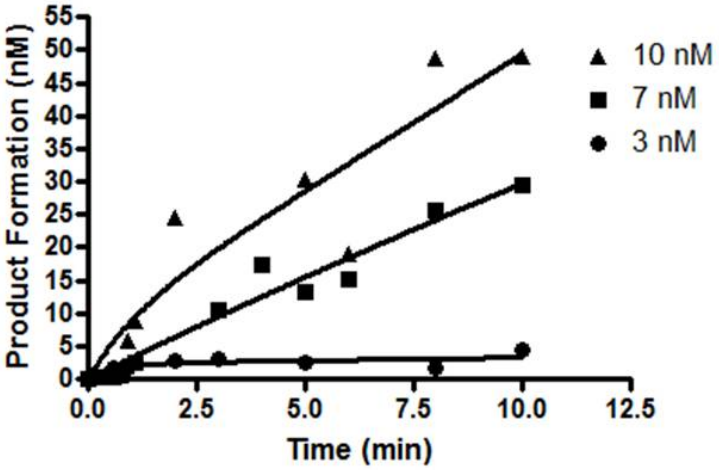

MPG* (Hx)	A_0	%
**10 nM**	**7.8**	**78.0**
**7 nM**	**6.5**	**92.8**
**3 nM**	**2.3**	**76.7**

* Preparation of purified MPG used for all Hx experiments
